# Purpura Fulminans and Septic Shock With Streptococcus pneumoniae Bacteraemia in a Patient With Unacknowledged Functional Hyposplenism

**DOI:** 10.7759/cureus.102299

**Published:** 2026-01-26

**Authors:** Christopher Ramos Huamancondor, Denis Glorieux

**Affiliations:** 1 Emergency Department, Cliniques Universitaires Saint-Luc (CUSL), Brussels, BEL; 2 Intensive Care Unit, Grand Hôpital de Charleroi, Charleroi, BEL

**Keywords:** disseminated intravascular coagulation (dic), fulminans purpura, hyposplenism, invasive pneumococcal disease, septic shock (ss), streptococcus pneumoniea

## Abstract

Purpura fulminans is a rare but devastating life-threatening condition characterised by a subtype of disseminated intravascular coagulation with extensive tissue thrombosis and hemorrhagic skin necrosis.

We report the case of a male patient in his 50s, with unacknowledged functional hyposplenism suspected after an abdominal CT scan on admission, who was admitted in septic shock secondary to *Streptococcus pneumoniae* bacteraemia. The patient’s condition deteriorated rapidly, leading to multiorgan failure, anuric acute kidney injury, disseminated intravascular coagulation with purpura fulminans, and encephalopathy. Management focused on the early initiation of dual antibiotic therapy, fluid resuscitation with crystalloids, hemodynamic support, renal replacement therapy, and mechanical ventilatory support. The patient survived but developed digital and lower limb necrosis, requiring multiple necrosectomies and skin grafts to optimise the outcome of bilateral transtibial amputations.

This case highlights the potential severity of purpura fulminans secondary to *Streptococcus pneumoniae* bacteraemia in a patient with functional hyposplenism.

## Introduction

*Streptococcus pneumoniae* (SP) is an encapsulated gram-positive, alpha-haemolytic bacterium that is a major cause of infection in children and older adults, which can cause infection of the respiratory tract (otitis media, sinusitis and pneumonia) or systemic disease; also called invasive pneumococcal disease (IPD) (bacteraemia, meningitis, endocarditis and arthritis) [1].

The main risk conditions for IPD in adults are immunocompromising conditions, including transplant recipients, asplenia, people living with human immunodeficiency virus, haematological malignancy, lower age or older > 65 years old, chronic kidney disease, compromised cerebrospinal fluid barrier and Down syndrome [2,3].

Purpura fulminans (PF) is a rare but devastating life-threatening condition characterised by a subtype of disseminated intravascular coagulation (DIC), with extensive tissue thrombosis and haemorrhagic skin necrosis [4]. There are three forms of purpura fulminans: neonatal, idiopathic and infection-associated, which is the most common form of purpura fulminans [4].

Infection-associated purpura fulminans is generally triggered by bacterial infections with encapsulated microorganisms such as *Neisseria meningitidis*, *Streptococcus pneumoniae*, *Haemophilus influenzae* or *Capnocytophaga canimorsus* [4,5]. Case reports of PF induced by *Streptococcus pneumoniae* are described but are uncommon in immunocompetent patients [3,6,7]. A retrospective French cohort study identified SP in only 21.9% of PF, far behind *Neisseria meningitidis* and meningococcemia, representing 63.7% of PF [8].

Moreover, of the 22% patients admitted to the intensive care unit (ICU) with *Streptococcus pneumoniae*-induced purpura fulminans, half of the cases occurred in asplenic or hyposplenic patients, with a sevenfold higher prevalence compared with eusplenic patients [9].

The pathophysiology is a failure of the anticoagulant protein C pathway, which leads to uncontrolled microvascular clotting with endothelial and cutaneous lesions because of inadequate protein C-mediated cytoprotective effects, which are vital for survival in sepsis [4,10,11]. The presence of bacteria within the cutaneous vascular wall, particularly *Streptococcus pneumoniae*, promotes the development of prothrombotic lesions [12].

Patients who survive past the first 24-72h often die from complications of unchecked thrombosis rather than shock, and survivors are usually left with severe scarring and tissue loss [4].

Management is based on the prompt initiation of broad-spectrum intravenous antibiotics, haemodynamic stabilisation, correction of coagulopathy, and, in selected cases, supplementation with protein C [4,10,12].

We report the case of a male patient in his fifties, with unrecognised functional hyposplenism, admitted to the intensive care unit from the emergency department for septic shock due to *Streptococcus pneumoniae* bacteremia, complicated by multiorgan failure and extensive purpura fulminans at presentation.

## Case presentation

A man in his fifties was admitted to the emergency department via the Mobile Medical Team (MMT) for respiratory distress and shock.

His medical history included non-insulin-dependent type 2 diabetes mellitus, nodular prurigo treated with phototherapy and arterial hypertension.

The patient had complained since the previous day of an unusual acute resting dyspnea associated with nausea, vomiting, diarrhoea and abdominal pain mainly localised to the left iliac fossa. His wife had recently experienced bronchitis, suggesting a possible infectious contact. There was no history of recent travel, prior antibiotic use, regular alcohol consumption or drug abuse. The patient’s vaccination status, particularly with regard to pneumococcal vaccination, was unknown at the time of admission.

On arrival of the Mobile Medical Team at his home, his vital signs were as follows: oxygen saturation 88% on room air, respiratory rate 20 breaths per minute, blood pressure 119/86 mmHg, tachycardia 130 beats per minute, temperature 39.6 °C, and Glasgow Coma Scale (GCS) of 14 E(4)V(4)M(6) with confusion. Initial management included oxygen therapy at 4 litres/minute, fluid resuscitation with 500 mL of crystalloids, and intravenous paracetamol 1 g.

During transport, his oxygen requirement increased to 6 litres/min, with persistent mottled skin despite fluid resuscitation. The patient was therefore transferred directly to the emergency resuscitation room.

Upon arrival, his vital parameters remained unchanged. The patient was admitted with septic shock based on an initial sequential organ failure assessment (SOFA) score of 14 points.

**Figure 1:**
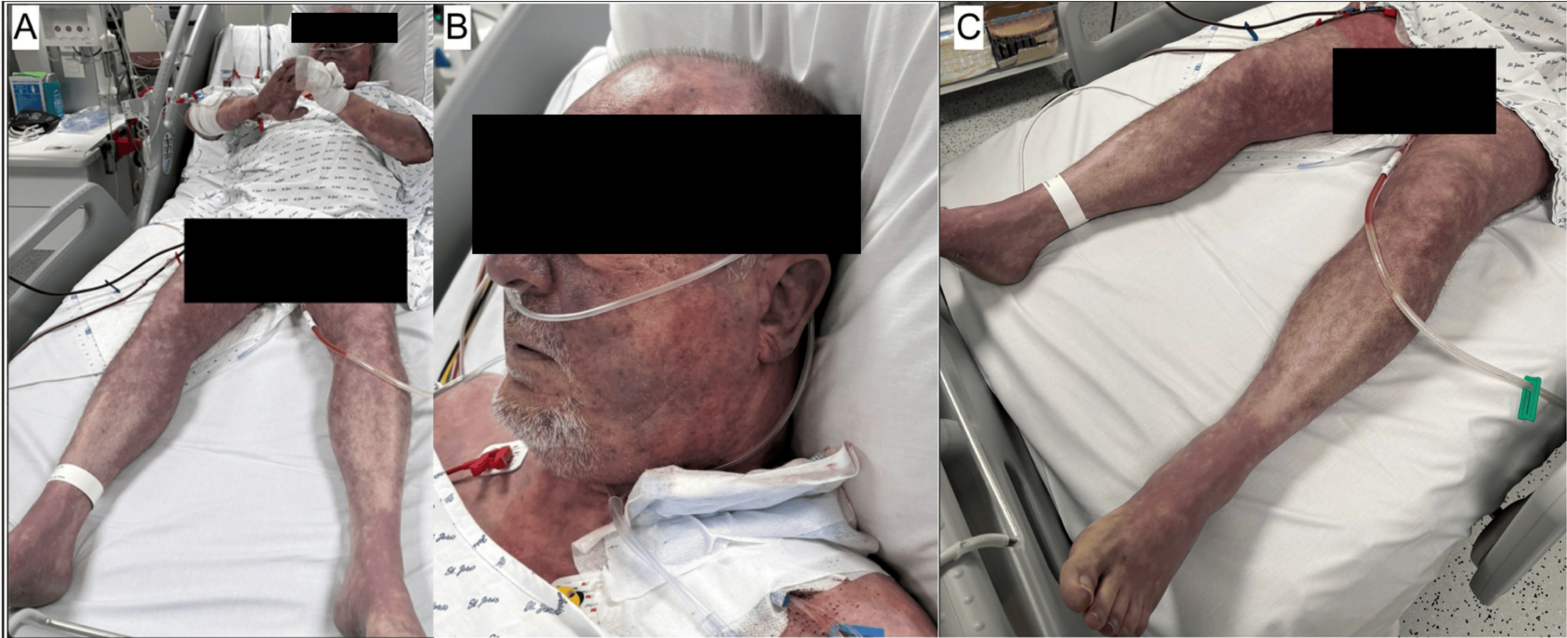
Extensive purpura fulminans at admission (A) Widespread violaceous purpura consistent with livedo racemosa, involving the trunk and face (B), as well as all four limbs, with prominent involvement of the lower extremities (C)

Clinical examination revealed diffuse mottling and acrocyanosis, with multiple violaceous, indurated lesions over the chest, abdomen, face, and all four limbs, consistent with purpura fulminans (Figure 1). The abdomen was soft, non-tender and there was no lower limb oedema.

Arterial blood gas analysis on admission revealed hypoxemic respiratory failure, with a partial pressure of oxygen (PaO₂) of 76 mmHg while receiving oxygen at 6 L/min, respiratory alkalosis with a pH of 7.5 and partial pressure of carbon dioxide (PCO2) of 19.4 mmHg, and elevated lactate at 8.98 mmol/L.

The admission electrocardiogram showed sinus tachycardia at 129 bpm with no ischaemic changes.

Initial blood tests demonstrated evidence of multiorgan failure consistent with septic shock, including a marked inflammatory response (C-reactive protein 310 mg/L), neutrophil-predominant leukocytosis, and regenerative haemolytic anaemia (haemoglobin nadir 7.3 g/dL at 72 hours after admission) with decreased haptoglobin (0.125 g/L) and the presence of schistocytes (Table 1).

**Table 1:** Laboratory values at admission Actin FS = in vitro diagnostic reagent for the quantitative determination of activated partial thromboplastin time (APTT) as an aid to diagnosis, screening for hemostasis disorders and monitoring of unfractionated heparin in human sodium citrated plasma by means of automated, semi-automated and/or manual coagulometric methods.

Laboratory value	Result	Reference Range	Unit
Haemoglobin (Hb)	13.8	12.6-17.3	g/dL
White cell count	32.5 ↑	4.0-11.0	x10^3^/mm^3^
Platelets	23 ↓	140-450	x10^3^/mm^3^
Creatinine	2.94 ↑	0.60 – 1.30	mg/dL
Urea	69 ↑	18 - 55	mg/dL
Aspartate aminotransferase (AST)	304 ↑	< 35	U/L
Alanine aminotransferase (ALT)	133 ↑	< 45	U/L
Total bilirubin	4.04 ↑ (max 16.06)	0.30 - 1.20	mg/dL
C-reactive protein (CRP)	321 ↑	0 - 5	mg/L
Prothrombin Time percentage activity (PTp)	36.6 ↓	70 - 100	%
International normalised ratio (INR)	2.88 ↑	1 - 1.5	-
activated partial thromboplastin time (aPTT) (actin FS) patient time	66.4 ↑	20.7 - 29.6	seconds
activated partial thromboplastin time (aPTT) (actin FS) control time	25.2		seconds
Patient/control (aPTT) ratio	2.63 ↑	0.82 - 1.18	-
Fibrinogen	0.98 ↓	2.00 - 4.00	g/L
Schistocytes	40 ↑	0 - 10	/1000 red blood cells
Haptoglobin	0.125↓	0.140 - 2.580	g/L
Lactate	9.13 ↑	0.33 - 1.20	mmol/L

Findings were also consistent with disseminated intravascular coagulation (DIC), with thrombocytopenia at 23,000/mm³ (nadir 10,000/mm³ at 48 hours after admission), an INR of 2.88 (prothrombin time percentage activity of 29.3%), and decreased fibrinogen at 0.98 g/L (D-dimers not measured).

Additionally, there was evidence of acute kidney injury (AKIN (Acute Kidney Injury Network) stage 3) with a serum creatinine of 2.94 mg/dL and mixed metabolic acidosis, as well as hepatic cytolysis and conjugated hyperbilirubinaemia (peak bilirubin 11.95 mg/dL on day 7 of admission).

Microbiological investigations performed in the emergency department included sputum culture, two pairs of blood cultures, urine culture, and urinary antigen testing, which was positive for Streptococcus pneumoniae. PCR testing for SARS-CoV-2 was negative. Owing to the patient’s condition and the high risk of bleeding, a lumbar puncture could not be safely performed.

**Figure 2:**
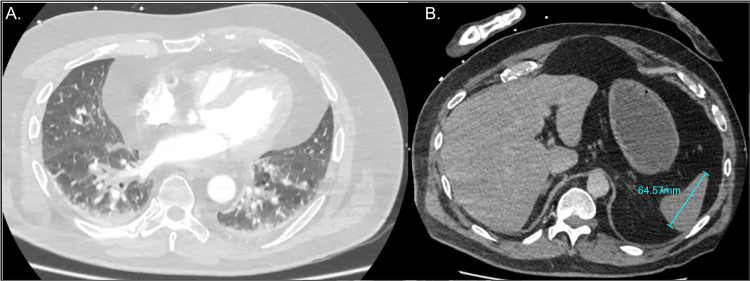
Total body scan at admission A. Axial chest CT scan showing non-specific ground-glass opacities associated with bibasal atelectasis. B. Contrast-enhanced abdominal CT demonstrating a small spleen measuring 64.57 mm in length.

Chest radiography revealed no pulmonary infiltrates. Contrast-enhanced thoracoabdominal CT imaging showed no deep-seated infectious focus, arterial dissection, pulmonary embolism, or mesenteric ischaemia. The only findings were a 6-mm right fissural pulmonary micronodule with ground-glass opacities and a markedly small spleen (6.4 × 6.0 × 5.1 cm) (Figure 2).

Therapeutic management followed the International Surviving Sepsis Campaign guidelines for septic shock. A second round of fluid resuscitation with crystalloids (total 2.5 litres) was administered, followed by vasopressor support with noradrenaline. Empirical antibiotic therapy with intravenous amoxicillin-clavulanate (1 g) was initiated within the first hour in the emergency department.

After transfer to the intensive care unit, the patient’s clinical condition deteriorated, with refractory hypotension and worsening hyperlactataemia. Toxic shock syndrome (TSS) was suspected, and antimicrobial therapy was escalated to include a single dose of amikacin (1.5 g or 30 mg/kg), meropenem (3 g), and clindamycin (900 mg). Intravenous hydrocortisone (100 mg three times daily) was added due to increasing vasopressor requirements. A trial of dobutamine was unsuccessful and subsequently discontinued.

Transthoracic echocardiography revealed preserved left ventricular ejection fraction, with no valvular abnormalities or pericardial effusion.

Both pairs of blood cultures taken in the emergency department turned positive after 3 hours and 30 minutes of incubation, showing Gram-positive cocci in chains. The organism was later identified as *Streptococcus pneumoniae*, with a minimum inhibitory concentration (MIC) of 0.5 µg/mL for penicillin and 0.094 µg/mL for cefotaxime (Table 2).

**Table 2:** Streptococcus pneumoniae susceptibility report Minimal inhibition concentration (MIC) breakpoints according to the European Committee on Antimicrobial Susceptibility 2023 (EUCAST) Sensitive (S), I = Intermediate (I) = Sensitive with a high dose regimen, Resistant (R)

Antibiotic	Susceptibility	MIC
Penicillin G	Intermediate	0.5 mcg/mL
Cefotaxime	Sensitive	0.094 mcg/mL
Meropenem	Sensitive	0.094 mcg/mL
Erythromycin	Resistant	N/A
Clindamycin	Resistant	N/A
Moxifloxacin	Sensitive	-
Tetracycline	Resistant	N/A
Trimethoprim-sulfamethoxazole	Sensitive	-

Following microbiological identification with antibiogram, antibiotic therapy was de-escalated to ceftriaxone 2 g twice daily for a total of 10 days.

The patient developed anuric acute kidney injury (AKIN stage 3), requiring continuous venovenous hemodiafiltration (CVVHDF) with citrate anticoagulation, initiated two hours after admission to the intensive care unit.

**Figure 3:**
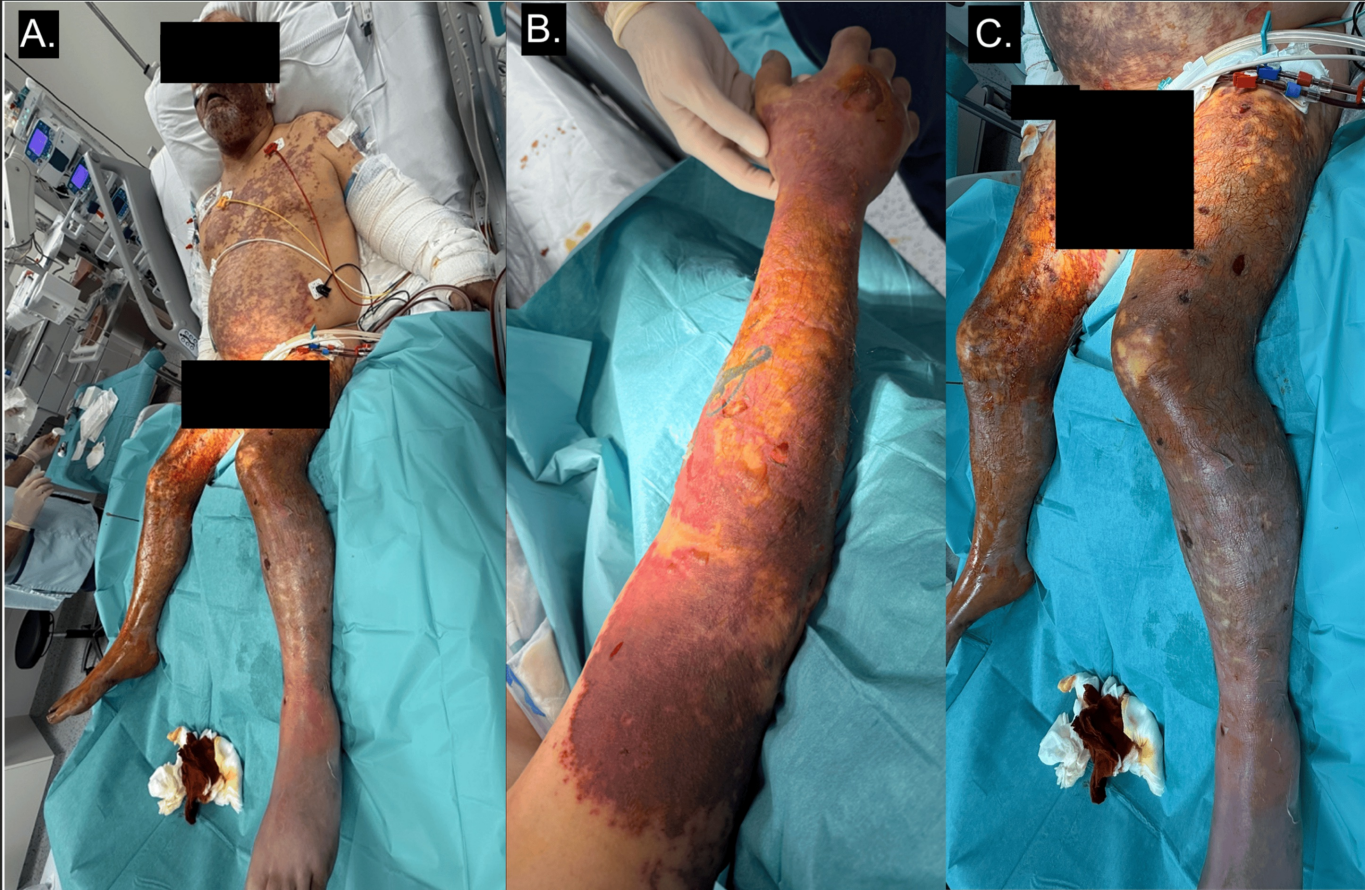
Extensive violaceous purpura 48 hours after admission to intensive care Involving the face, chest, abdomen and all four limbs (A). Progression of purpura to epidermal necrolysis with extensive blistering on the right upper limb (B) and lower limbs (C).

The extensive purpura fulminans involving all four limbs and the trunk at presentation progressed to bullae formation (Figure 3), followed by digital necrosis despite optimisation of haemodynamic parameters. Septic thrombocytopenia required three platelet pool transfusions in total but was not complicated by bleeding or external haemorrhage. ADAMTS13 activity was negative, excluding autoimmune thrombotic thrombocytopenic purpura. Cryoglobulin testing performed in the emergency department was also negative.

Oxygen requirements did not increase, but the patient required intubation six days after admission for analgesia and deep sedation to facilitate wound care.

The evaluation of Streptococcus pneumoniae bacteraemia included transthoracic echocardiography, which showed no valvular vegetations, fundoscopy without abnormalities, and a brain CT scan demonstrating only diffuse sinusitis without indication for drainage.

**Figure 4:**
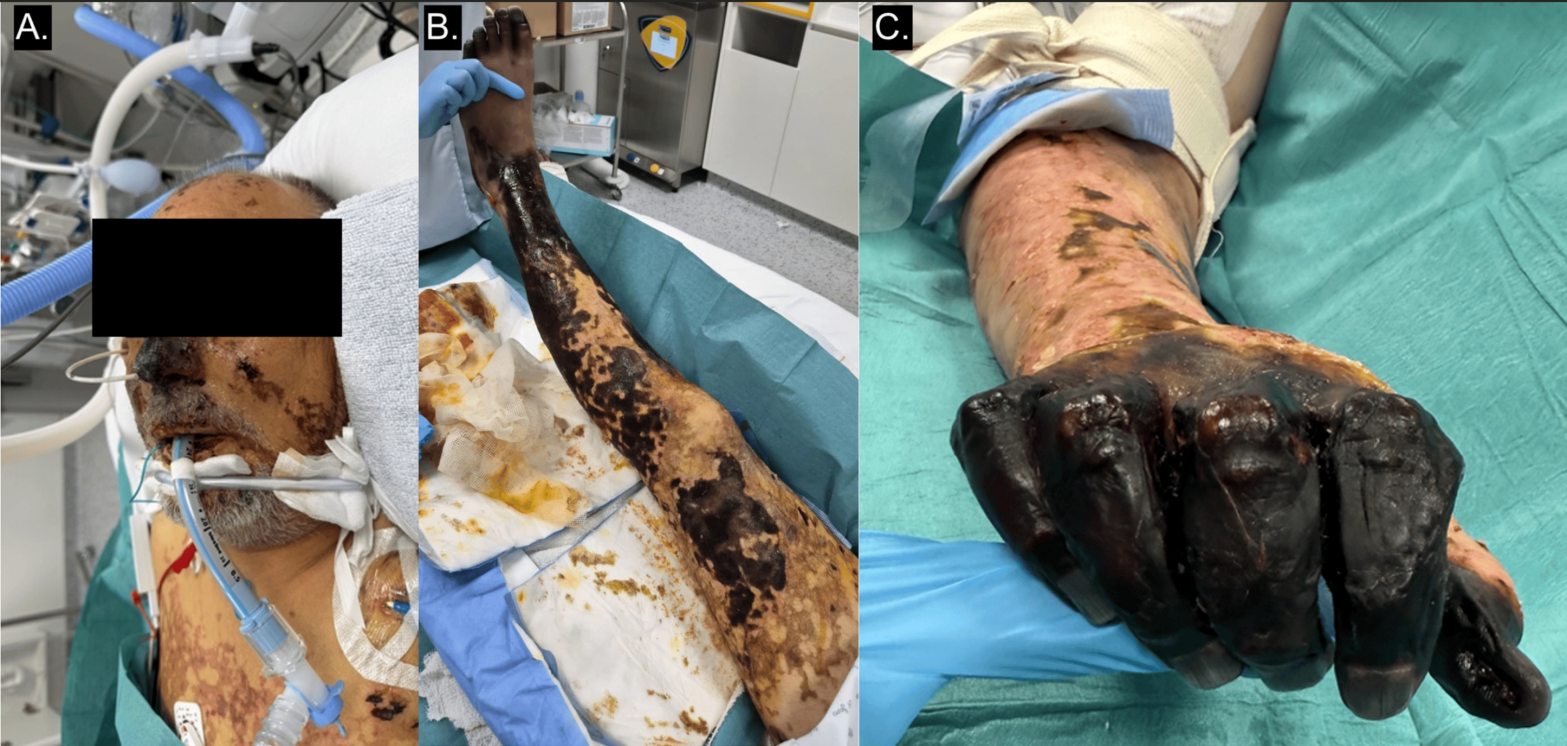
Evolution of purpura during the intensive care unit stay (A) Face: progression of purpura to dry nasal necrosis 11 days after admission(B) Right lower limb: superficial necrotic areas and dry digital necrosis (mummification) 11 days after admission (C) Right hand: complete necrosis of all digital extremities 40 days after admission

The final diagnosis was septic shock due to Streptococcus pneumoniae bacteraemia in an immunocompetent patient with a small spleen, consistent with probable functional hyposplenism, complicated by multiorgan failure and, notably, purpura fulminans progressing to dry necrosis of the extremities (Figure 4).

Although the patient survived, he unfortunately developed complications because of extensive necrotic skin lesions in his extremities due to the infectious purpura fulminans.

A multidisciplinary consultation with the burns unit was held, including a plastic surgeon, vascular surgeon, dermatologist, and orthopaedic surgeon, following vascular assessment with contrast-enhanced CT angiography of the lower limbs. Given the poor condition of the skin, the patient underwent several necrosectomies and multiple homo- and allografts to optimise the outcome of bilateral transtibial amputations.

Unfortunately, because of extensive necrosis of the fingers on both hands, bilateral transmetacarpal amputation was required.

After 50 days in the intensive care unit, the patient survived and was transferred to a specialist burns centre for ongoing management of the cutaneous necrosis. Due to his hyposplenism, the patient received preventive vaccination with a pneumococcal 20-valent polysaccharide conjugate vaccine, *Haemophilus influenzae* vaccine, and meningococcal (A and B groups) vaccine.

## Discussion

We report the case of a male patient in his fifties, initially considered immunocompetent but with possible functional hyposplenism, who developed septic shock due to *Streptococcus pneumoniae* bacteraemia, likely of pulmonary origin. The infection was complicated by severe anuric acute kidney injury and extensive purpura fulminans, leading to distal necrosis. The patient survived but required multiple necrosectomies and skin grafts to optimise the outcome of bilateral transtibial amputations.

Purpura fulminans (PF) is a rare but devastating complication of sepsis, characterised by disseminated intravascular coagulation (DIC) and microvascular thrombosis of the skin, resulting in extensive tissue necrosis [4,10-14]. Although classically associated with *Neisseria meningitidis*, several recent reports describe pneumococcal purpura fulminans, particularly in asplenic (anatomical or functional) or immunocompromised patients [3,15-18], or with unrecognised underlying immunodeficiency [3,6].

For example, cases have also been reported in apparently immunocompetent, non-splenectomised patients, as described by McDonnell et al. and Djurdevic et al. [6,7]. Most of whom were unvaccinated, highlighting the importance of pneumococcal vaccination to prevent invasive pneumococcal disease (IPD).

This emphasises the crucial role of the spleen in host defence against encapsulated bacteria through phagocytosis, IgM production, and opsonisation [16,17].

The spleen regulates B cell function, allowing direct encounter with blood-borne pathogens captured by marginal zone macrophages and triggering the immediate recruitment of memory B cells [19]. Moreover, IgM memory B cells act as the first-line defence against infections, especially encapsulated bacteria polysaccharides, by secreting natural IgM and generating most of the IgA plasma cells at mucosal sites, such as the surface of epithelial cells of the airways, gut and genitourinary tract [19]. This explains why hyposplenism, defined by dysregulation of immune responses and alteration of blood filtration, exposes patients to these invasive bacterial infections [20]. There are many etiologies of hyposplenism, including haemotological, infectious (HIV), autoimmune and gastrointestinal disorders [19,20]. The diagnosis is based through either radioisotopic methods (abandoned because of impractical in clinical setting) or by searching for erythrocyte morphological alterations on blood smear showing a blood Howell-Jolly bodies (red cells with nuclear remnants), acanthocytes (red cells with spiked cells membrane), spherocytes (sphere-shaped red cells), stomatocytes and pitted red cell (PRC) count > 4% and/or IgM memory B cell count < 26/mcL [19].

Unfortunately, these tests were not carried out with our patient. However, we maintain a level of clinical suspicion based on the association of an invasive pneumococcal disease and a smaller spleen (6x5cm), compared to the mean adult length normal spleen at 8-12 cm [19]. Moreover, it is important to note that diabetes mellitus is also an independent risk factor for invasive bacterial infections [21,22]. Hyperglycemia, innate immune cell dysfunction, and endothelial cell damage are diabetes-related complications that can worsen bacterial infections, particularly soft tissue infections [21], leading to severe tissue necrosis in this case.

The physiopathology of infection-associated purpura fulminans involves a profound systemic inflammatory response to pneumolysin, a toxin produced by *Streptococcus pneumoniae*, which activates the coagulation cascade and leads to consumption of natural anticoagulant proteins, especially protein C [4]. Acquired protein C deficiency (typically <40%) contributes to the prothrombotic process responsible for the purpuric lesions and subsequent distal necrosis [4,5,12]. In most reported cases, this is associated with severe DIC, tissue hypoperfusion, and multiorgan failure.

Clinically, purpura fulminans presents as rapidly progressive purpuric lesions with a retiform or livedoid appearance, often affecting the extremities and associated with septic shock [4]. Progression to extensive necrosis and limb amputation is frequent despite early antibiotic therapy and haemodynamic support [6,16,23]. Reported mortality rates range from 40% to 60%, particularly high in cases of refractory shock or multiorgan failure [3].

Management focuses on the underlying cause and follows standard septic shock protocols, including early administration of broad-spectrum antibiotics (third-generation cephalosporins or amoxicillin/clavulanate ± vancomycin or clindamycin), haemodynamic and respiratory support, and corticosteroids [14,24,25].

Regarding antibiotic therapy, the patient initially received a single dose of amoxicillin/clavulanate in the emergency department based on the initial presumed diagnosis of severe community-acquired pneumonia (CAP IV or sCAP) in the context of comorbid diabetes mellitus.

Upon ICU admission, due to the lack of clinical improvement following the initial dose of amoxicillin-clavulanate, the need for increasing doses of norepinephrine, and the presence of severe refractory septic shock, antimicrobial therapy was escalated from amoxicillin-clavulanate to meropenem. This decision was made due to concern for septic shock caused by extended-spectrum β-lactamase (ESBL)-producing bacteria, and a single dose of amikacin was added.

Because initial blood cultures identified a *Streptococcus* species without further specification, clindamycin was added due to suspicion of Streptococcal toxic shock syndrome.

Forty-eight hours after ICU admission, once *Streptococcus pneumoniae* was identified with a complete antimicrobial susceptibility profile (Table 2) from the same blood cultures, clindamycin was discontinued, and antimicrobial therapy was de-escalated from meropenem to ceftriaxone for a total duration of 10 days, in accordance with current recommendations [25,26].

Toxic shock syndrome is defined as an exotoxin-mediated shock caused, most commonly, by group A *Streptococcus* (*Streptococcus pyogenes*) or *Staphylococcus aureus* [27,28], usually requiring empirical dual antibiotherapy with clindamycin (900 mg IV every 8 hours) plus carbapenem or penicillin with a beta-lactamase inhibitor (ticarcillin/clavulanate or piperacillin/tazobactam) or vancomycin [24,27,28]. Although clindamycin’s anti-toxinic effect is recommended in TSS or necrotising soft tissue infection (NIST) because it may significantly inhibit exotoxin production from Gram-positive pathogens and improve outcome [27], there are no data for *Streptococcus pneumoniae*-induced purpura fulminans.

The treatment of DIC is more complex and depends on the clinical stage [4,12,13]. The first step is to identify a significant protein C deficiency (<40%) and other coagulation abnormalities [4]. Some authors advocate the administration of protein C and antithrombin concentrates, along with correction of fibrinogen and platelet deficits (>30 × 10⁹/L) [4]. Subsequently, if clinically feasible, unfractionated heparin, fresh frozen plasma, and vitamin K may be considered [4]. Although such protocols aim to limit thrombotic progression and reduce the need for amputation, evidence from randomised controlled trials remains lacking. It should also be noted that sepsis-associated DIC typically manifests with a bleeding tendency rather than widespread thrombosis, with microthrombi observed in only 5-10% of cases [10].

All published cases underscore the critical importance of prevention through pneumococcal vaccination, which remains the most effective measure to prevent invasive pneumococcal infections, particularly in splenectomised, hyposplenic, or immunocompromised patients [8,9]. Current recommendations include sequential administration of conjugate (PCV13) and polysaccharide (PPV23) vaccines, in combination with immunisation against *Neisseria meningitidis*, *Haemophilus influenzae* type b, and seasonal influenza [29,30].

## Conclusions

Streptococcus pneumoniae can cause purpura fulminans, a rare but life-threatening complication, even in patients initially considered immunocompetent, particularly in the presence of unrecognised functional hyposplenism. Functional or anatomical asplenia is a major risk factor for invasive pneumococcal disease, and clinicians should actively consider hyposplenism in patients presenting with severe pneumococcal sepsis, especially when associated with disproportionate disease severity. Purpura fulminans is driven by sepsis-induced dysregulation of coagulation, notably acquired protein C deficiency and disseminated intravascular coagulation, leading to extensive microvascular thrombosis and tissue necrosis.

Early recognition and aggressive multidisciplinary management are essential, including prompt broad-spectrum antibiotics, haemodynamic support, and careful management of coagulation abnormalities, although evidence for adjunctive anticoagulant therapies remains limited. Prevention through appropriate vaccination is crucial, as pneumococcal immunisation remains the most effective strategy to reduce the incidence and severity of invasive pneumococcal infections, particularly in splenectomised, hyposplenic or otherwise at-risk patients.
